# Precuneus activation correlates with the vividness of dynamic and static imagery: an fMRI study

**DOI:** 10.3389/fnhum.2025.1516058

**Published:** 2025-03-14

**Authors:** Suna Duan, Qingfeng Li, Junjie Yang, Qing Yang, Enran Li, Yuting Liu, Lijuan Jiang, Chunbo Li, Binglei Zhao

**Affiliations:** ^1^Shanghai Mental Health Center, Shanghai Jiao Tong University School of Medicine, Shanghai, China; ^2^School of Psychology, Shanghai Jiao Tong University, Shanghai, China; ^3^School of Biomedical Engineering, ShanghaiTech University, Shanghai, China

**Keywords:** static imagery, dynamic imagery, precuneus, functional magnetic resonance imaging, vividness of visual mental imagery

## Abstract

**Introduction:**

Visual mental imagery (VMI) is a cognitive function that significantly impacts various aspects of daily life. However, the neural correlates of VMI vividness remain unclear, especially underlying different VMI types. Therefore, the current functional magnetic resonance imaging (fMRI) study aimed to investigate the neural mechanisms underlying static (SI) and dynamic VMI (DI), focusing on the role of precuneus especially in the imagery vividness.

**Methods:**

The study involved 24 participants recruited from ShanghaiTech University. After excluding four participants due to excessive movements, data from 20 participants were analyzed. Each participant completed the Chinese version of the Vividness of Visual Imagery Questionnaire (VVIQ) to assess their imagery vividness abilities. During fMRI scanning, participants were asked to imagine SI and DI scenarios in response to auditory stimuli. High-resolution fMRI data were acquired using a 3T scanner, and a General Linear Model (GLM) was applied to analyze blood oxygenation level-dependent (BOLD) signals, focusing on the precuneus’s role in imagery vividness.

**Results:**

The results revealed that the left precuneus was found activated in both SI and DI tasks. Moreover, the left precuneus activation was positively correlated with VVIQ score. On the other hand, greater activation in the right precuneus was found during dynamic than static imagery as well as more extensive neural engagements; the right precuneus activation was further detected significantly correlated with individual VVIQ scores.

**Discussion:**

The study’s findings offered fresh insights into the cognitive and neural processes subserving VMI. By revealing the distinct roles of the left and right precuneus in imagery vividness, this research contributed to a more nuanced understanding of VMI and its neural basis.

## 1 Introduction

Visual mental imagery (VMI) refers to the perception-like representations that people form in their minds when things are not in sight, like a weak form of perception (Pearson, 2019). VMI plays an essential role in our daily life and affects lots of cognitive functions, such as episodic memory (D'Angiulli et al., 2013), spatial navigation (Bird et al., 2012; Carbone et al., 2021), reading comprehension (De Koning and van der Schoot, 2013; Leutner et al., 2009), creativity (Benedek et al., 2020; May et al., 2020) and moral decision-making (Amit and Greene, 2012). Though recent studies have shed light on the brain areas activated during imagination (c.f., Fulford et al., 2018; Hajhajate et al., 2022; Liu and Bartolomeo, 2023a; Winlove et al., 2018), the neural mechanisms underlying different types of VMI are still not fully understood.

There are at least two types of VMI, to represent a static object or scene (*static imagery*, SI) or the dynamic manipulation process of mental images (*dynamic imagery*, DI) (c.f., Paivio and Clark, 1991; Pearson, 2019). Some patients have been reported to be able to imagine the characteristics of static objects, such as color and shape, but to have difficulty in mentally rotating them (Aleman et al., 2005; Luzzatti et al., 1998). In contrast, other patients can mentally rotate objects but cannot visualize static objects (Karnath et al., 2009; Lê et al., 2002). These results seem to reveal a dissociable association between SI and DI processes (i.e., Aleman et al., 2005; Borst, 2013; Levine et al., 1985). Thanks to the neuroimaging technology, the neural mechanisms can be further explored and uncovered different brain activation patterns underlying SI and DI (i.e., Dijkstra et al., 2019). In a series of functional magnetic resonance imaging (fMRI) studies, SI was found accompanied by the activation of the precuneus, superior parietal cortex, prefrontal cortex and several regions in the occipito-temporal cortex when participants were required to represent an apple or a forest in their minds’ eyes (Dijkstra et al., 2019; Ganis et al., 2004; Ishai et al., 2000; Kosslyn et al., 2001; Mechelli et al., 2004; Winlove et al., 2018). Different brain areas (i.e., premotor areas, parietal areas, prefrontal cortex and precuneus) were observed to active in DI tasks (Bien and Sack, 2014; Burianová et al., 2013; Formisano et al., 2002; Hétu et al., 2013; Kosslyn et al., 2001; Parsons et al., 1995; Wang et al., 2023; Wang et al., 2020) when participants were required to subjectively imagine a motor process in minds (i.e., climbing stairs; Cui et al., 2007) or to complete tasks that required mental manipulation (i.e., dynamically rotating an actual physical object in mental rotation tasks; Shepard and Metzler, 1971). These brain regions also played a crucial role in tasks involving motor imagery of grasping, where individuals imagined the complex motor actions required to grasp and manipulate objects. This process engaged the anterior intraparietal area, ventral premotor cortex, dorsal premotor cortex, and the supplementary motor area (Bencivenga et al., 2021, 2023). However, to our best knowledge, there was no neuroimaging study yet directly comparing SI and DI neural mechanisms.

In addition, vividness stands out as a key feature in imagery processes (Fulford et al., 2018). Vividness of visual imagery (VVI) refers to the clarity and richness of details in the visual image one can generate in the mind (Marks, 1973; Hishitani and Murakami, 1992). Vividness of Visual Imagery Questionnaire (VVIQ) (Marks, 1973; Zhang et al., 2024) is the most widely used measurement for such abilities (Cui et al., 2007; Fulford et al., 2018; Liu and Bartolomeo, 2023b). This questionnaire comprises four scenarios related to VMI, covering four domains: color, detail, depth, and movement. Participants were asked to imagine each scenario and to rate the vividness of their imagination on a five-point scale using the Likert scale (1, no image to 5, very vivid) (Marks, 1973; Zhang et al., 2024). VVI abilities were found to vary across individuals: some can generate very vivid mental images in minds that are truly as lively and vivid as real seeing while others may fail to generate or can create very vague images (Zeman et al., 2015; Keogh et al., 2021).

The individual difference in VVI abilities was observed to affect both SI (Dijkstra et al., 2017; Fulford et al., 2018) and DI processes (Cui et al., 2007; Logie et al., 2011; Zhao and Sala, 2018; Zhao et al., 2019; Zhao et al., 2022). Systematic differences between individuals with higher and lower VVI abilities were observed in SI while famous faces or places were required to be generated: the posterior visual network was activated in higher imagers whereas the frontal network was observed activated in lower imagers, including the inferior frontal and anterior cingulate gyrus (Fulford et al., 2018). Moreover, the connectivity strength between the occipital place area and the parahippocampal place area correlated positively with an individual’s VVIQ score, indicating a link between brain region connectivity and the vividness of visual imagery (Tullo et al., 2022). In coping with mental rotation tasks (Shepard and Metzler, 1971), a classic measurement for DI, differential brain activation patterns were found between individuals with lower and higher VVI (Logie et al., 2011).

Notably, the key brain areas responsible for VVI abilities are still unclear, though more researchers shed light on the neural mechanisms of VMI (c.f., Bartolomeo et al., 2020; Spagna et al., 2021; Winlove et al., 2018). The precuneus is somehow neglected though always reported activation in VMI tasks (Fulford et al., 2018; Mazzoni et al., 2019; Winlove et al., 2018). For example, Zvyagintsev et al. (2013) reported the activation of the left precuneus when participants were asked to imagine familiar static objects (i.e., animals or trees). Similar observations were observed in SI tasks accompanied by the activation in bilateral precuneus (de Borst et al., 2012; Gardini et al., 2009). In addition, the left precuneus was also found to be activated when participants were asked to perform DI tasks (i.e., mental rotation task) (Podzebenko et al., 2002). When participants were asked to imagine a movement (i.e., pushing a door), the precuneus was found to activate as well as other brain areas (Confalonieri et al., 2012; Hanakawa et al., 2003). Interestingly, precuneus activation was also reported when VVI abilities were taken into account (i.e., Fulford et al., 2018; Dijkstra et al., 2017). For example, when participants were required to imagine famous faces or places (e.g., Einstein), the left and right precuneus has been reported positively correlated with individual VVI abilities (Fulford et al., 2018; Dijkstra et al., 2017).

Based on the existing observations, therefore, we proposed that precuneus would play a key role in VVI abilities. To test this hypothesis, all participants were assessed with SI and DI tasks in the magnetic resonance scanner while functional images with blood oxygenation level-dependent (BOLD) contrast were acquired. Their imagery vividness abilities were assessed with VVIQ (Marks, 1973; Zhang et al., 2024). First, the brain activation patterns were explored in SI and DI processes, respectively. To address the difference between these two imagery types, brain activations were compared between SI and DI processes in all imagers. In addition, the association between the bilateral precuneus and VVI abilities was indexed by VVIQ scores. Gleaned from the literature, we predicted that both left and right precuneus would be activated in both SI and DI. Considering the imagery task complexity (i.e., how much vivid information should be represented in minds), there would be distinctive neural mechanisms between SI and DI, especially in precuneus if it is the key area for VVI abilities. In addition, there would be correlations observed between precuneus and VVI abilities in SI and DI processes.

## 2 Materials and methods

### 2.1 Participants

Twenty-four participants from ShanghaiTech University were recruited (four females, ages 19–37, *M* = 22.79 years old, SD = 4.27 years old). All participants were right-handed, had no history of physical or mental illness, and had normal or corrected-to-normal vision. Additionally, participants scored higher than 32 points on the Visual Image Vividness Questionnaire (VVIQ) to ensure that our sample consisted of individuals with visual image abilities (Marks, 1973). The experimental design was approved by the Ethics Committee of Shanghai Jiaotong University (ethical no.: H20230182I), and all participants read and signed the fMRI experiment informed consent before the experiment. Four participants were excluded from the analysis due to head movements exceeding our predefined criteria for excessive motion: translations greater than 3 mm and rotations greater than 3 degrees. Thus, the remaining 20 participants’ data (three females, ages 19–27, *M* = 22.00 years old, SD = 2.51 years old) were used for data analysis. None of these participants participated in our previous fMRI study of imagery. To determine the adequacy of our sample size, a power analysis was performed using G*Power 3.1 (Faul et al., 2009; Faul et al., 2007). Assuming an effect size of 0.5, alpha level of 0.05 and an expected power of 0.80 (Cohen, 1988; Spagna et al., 2021; ZHENG et al., 2011). For a within-subjects design, G*Power indicated that a sample size of 20 provides adequate power to detect medium to large effects in our analyses.

### 2.2 Subjective vividness rating

Each participant completed the Chinese version of the Vividness of Visual Imagery Questionnaire (VVIQ-C; Zhang et al., 2024). Similar to Marks’s (1973) original version, the VVIQ-C consists of 16 items to measure the vividness of the participants’ mental imagery. Participants were asked to create a mental image of a specific scene or object (e.g., the rising sun and familiar relatives) and to rate its vividness on a 5-point Likert scale, 1 means no image at all, 5 means the image is very vivid and clear. Total scores range from 16 to 80.

### 2.3 Experimental task

Static (SI) and dynamic imagery (DI) auditory stimuli were designed in the present study. In the SI trails, participants were instructed to imagine either “familiar relatives” or “dense forest.” In the “familiar relatives” trial, participants were instructed to close their eyes, listen to the phrase “familiar relatives” and visualize in their minds their close family members, such as parents, siblings, or close friends. They were asked to imagine the physical characteristics of these individuals and other static visual images in as much detail as possible. In the “dense forest” trial, participants were required to imagine various static object details within the forest (i.e., the texture of the trees). Two auditory cues were involved in DI trials, “pouring rain” or “climbing stairs” in which motor imagery were engaged (Cui et al., 2007; Munzert et al., 2009; Szameitat et al., 2007). In the “pouring rain” trial, participants were instructed to imagine a scene of “pouring rain,” where they needed to mentally construct a dynamic scene with rainwater cascading from the sky. For the “climbing stairs” trial, participants were asked to visualize themselves in the dynamic process of climbing stairs. Each stimulus was presented with five Chinese characters, which were converted into auditory stimuli through professional text-to-auditory software to ensure that the stimulus duration (all within 2 s), pronunciation, and volume were consistent.

**Figure 1:**
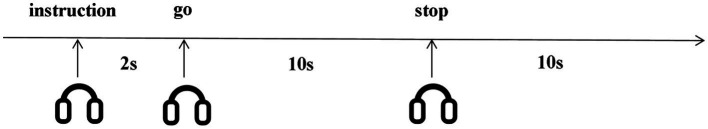
Imagery task timeline. Participants started visualization after hearing the ‘go’ signal, and stopped visualization when they heard ‘stop’. Participants visualized for 10 seconds, rested for 10 seconds. All instructions were auditory.

The experimental procedure was similar to Cui et al.’s (2007) design. An eye patch was given to each participant and they were instructed to close their eyes for the entire experiment. At the beginning of each trial (as shown in Figure 1), an auditory instruction was given lasting for 2 s prompting participants to imagine either an object or an action. To ensure imagery starts at the same time, participants were instructed to start visualization after hearing the ‘go’ signal and they had 10 s to imagine (imagery phase). They were instructed to stop visualization and had a 10-s rest when they heard ‘stop’ before the next trial. This rest period allowed the BOLD signal to return to baseline. Each auditory stimulus was presented eight times in a random order for each subject. Therefore, there were 32 trials (4 types of auditory stimuli × 8 repetition 32 trials) in total.

Upon completion of the experiment, a debriefing session was conducted with each participant to verify whether they had engaged in mental imagery as required by the tasks. Specifically, each participant was asked to confirm whether they had performed mental imagery during the experimental trials. All participants confirmed that they had indeed engaged in imagery tasks as instructed.

### 2.4 MRI acquisition

Images were acquired using a 3T UIH uMR790 MR whole-body scanner (United-Imaging Healthcare, China). High-resolution anatomical images were acquired using a 3D T1-weighted Gradient-Recalled Echo (GRE) sequence (TR: 8.1 ms, TE: 3.4 ms, voxel size: 0.8 mm × 0.8 mm, thickness: 0.8 mm, number of slices: 208, field of view: 256 mm × 240 mm × 208 mm, flip angle: 8°). Functional images were obtained using an Echo-Planar Imaging (EPI) sequence (TR: 2000 ms, TE: 30 ms, voxel size: 3.5 mm × 3.5 mm, thickness: 4 mm, number of slices: 33, field of view: 224 mm × 224 mm × 132 mm, flip angle: 90°, number of volumes: 352). Slices were acquired in the axial plane, parallel to the anterior commissure/posterior commissure (ACPC) line.

Quality control measures were implemented during the study to ensure data integrity, including regular assessments using water phantoms to verify device stability. During data collection, scanning technicians rescanned participants if excessive head motion was detected (monitored in real time by United Imaging’s mocap system) or if artifacts were present in the reconstructed images. Additionally, during the data analysis phase, participants were excluded if their head movements exceeded predefined criteria for excessive motion, specifically translations greater than 3 mm and rotations greater than 3 degrees.

### 2.5 fMRI data processing

fMRI preprocessing and statistical analysis were performed using MATLAB R2019b (The MathWorks, Inc., Natick, MA) and SPM12 (Statistical Parametric Mapping software).^1^ For each fMRI image, we first performed slice timing correction, then spatially realigned the images to the reference volume (i.e., the first acquired volume) and then co-registered to the mean EPI image. The mean EPI image was normalized to the standard single subject template in MNI space. A Gaussian kernel of 4 mm full-width half-maximum was used for smoothing to meet the statistical requirements of the theory of Gaussian fields according to the General Linear Model employed in SPM and to compensate for inter-individual variability in macro- and micro-anatomical structures across subjects (Friston et al., 1995a, b).

A General Linear Model (GLM) was thus applied to each voxel of the functional dataset (i.e., first-level analysis). We used an event-related analysis and the BOLD response for each event type was modeled with the canonical Hemodynamic Response Function (HRF) and its temporal derivative. A temporal high-pass filter of 1/128 Hz and linear trend removal were employed. The three translations and the three rotation movement parameters obtained from the initial spatially realignment were included as further regressors.

For this experiment, two event types were defined and then used as conditions for the model specification: (a) dynamic imagery, “DI,” (b) static imagery, “SI.” To assess specific effects, we applied appropriate linear contrasts of the parameter estimates for the DI and SI conditions. For each participant, we calculated the following contrast images: DI > Rest and SI > Rest, which represent the activation patterns specific to each imagery condition compared to the resting state. We also calculated the main effect contrasts (DI > SI and SI > DI) to directly compare the two imagery conditions. Second level Random Effects Analyses were performed by using a *t*-test to create an SPM{T} on contrast images obtained from individual participants, to obtain significant activations specific for each contrast on a group level (i.e., second-level analysis). We used a threshold of *p* < 0.05, corrected for multiple comparisons at the cluster level using family-wise error (FWE), with a height threshold at the voxel level of *p* < 0.05, uncorrected. The xjView Toolbox v10 and the Anatomical Automatic Labeling (AAL) atlas were employed for the anatomical localization and labeling of activation clusters in the brain (Tzourio-Mazoyer et al., 2002). The code for the first-level and second-level analyses can be found on this page.^2^

### 2.6 Behavioral data analysis

VVIQ data was analyzed using the SciPy v1.7.3 toolkit of Python 3.7. Group-level correlation between VVIQ scores and brain activations was calculated by computing the Pearson correlation coefficient, in which brain activation of certain ROI was calculated by averaging the voxel intensity of the contrast images (generated in first-level analysis) covered by the ROI.

## 3 Results

### 3.1 The VVIQ score of the participant

The participants’ scores on the Vividness of Visual Imagery Questionnaire (VVIQ-C) ranged from 39 to 80, with an *M* score of 62.85 and a SD of 12.99.

### 3.2 Activation of brain regions in DI and SI task

**Figure 2:**
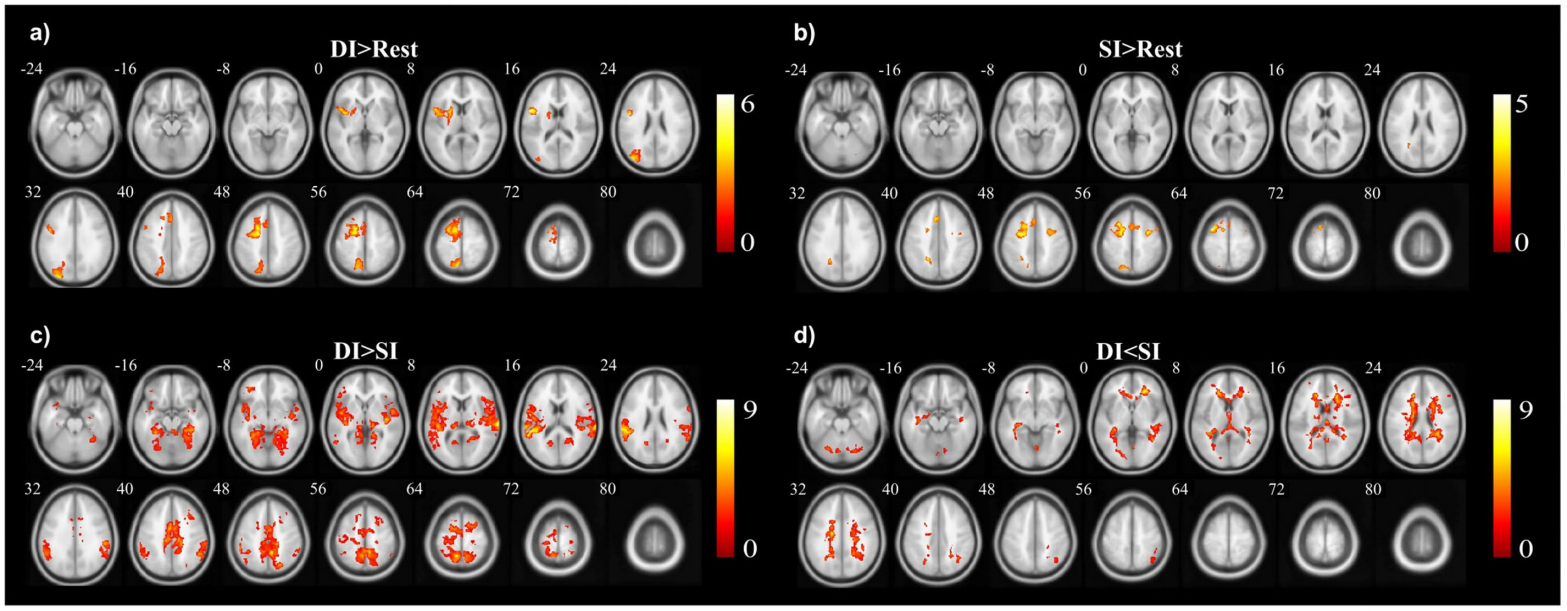
The activation clusters in the **(a)** dynamic imagery (DI) contrast (DI > Rest), **(b)** static imagery (SI) contrast (SI > Rest), **(c)** dynamic imagery > static imagery contrast (DI > SI), and **(d)** static imagery > dynamic imagery contrast (SI > DI).

A two-sample *t*-test was used to see the activated brain area in the DI task and SI task. The DI task engaged a network of brain regions. Specifically, the task-related network included the left inferior frontal gyrus, left precuneus, left supplementary motor area, left insula, left superior parietal lobe, and left superior frontal cortex (All cluster sizes >30 voxels, *p* < 0.05, cluster-wise FWE corrected; see Table 1; Figure 2a). The SI task also activated a distinct set of brain regions, including the right cerebellum crus1 lobule, left medial superior frontal cortex, middle frontal gyrus (bilaterally), left superior parietal lobe, and left supplementary motor area, along with the left precuneus (All cluster sizes >30 voxels, *p* < 0.05, cluster-wise FWE corrected; see Table 2; Figure 2b). The activation of the precuneus in both DI and SI tasks underscored its potential role as a common neural substrate for visual mental imagery, irrespective of the content of the imagery (DI and SI).

**Table 1:** Activation of brain regions in dynamic imagery (DI) task.

Anatomical area	Hemi	Co-ordinates			K	*t*-value
		X	Y	Z		
Inferior Frontal Gyrus	L	−52	8	14	1,346	6.84
Precuneus	L	−6	−62	68	1,795	6.46
Supplementary Motor_Area	L	18	−15	−18	2,457	5.62

Hemi, Hemisphere activation present in-left (L) or right (R). K, Cluster size. *t*-value, peak *t*-value.

**Table 2:** Activation of brain regions in static imagery (SI) task.

Anatomical area	Hemi	Co-ordinates			K	*t*-value
		X	Y	Z		
Cerebellum_Crus1	R	46	−58	−30	402	4.66
Undefined		−18	−52	40	408	3.59
Medial Frontal Lobe	L	−6	24	42	429	3.71
Middle Frontal Gyrus	R	30	−4	52	363	4.16
Middle Frontal Gyrus	L	−30	4	64	1,171	5.24

Hemi, Hemisphere activation present in-left (L) or right (R). K, Cluster size. *t*-value, peak *t*-value.

### 3.3 Activation differences between DI and SI tasks

When comparing the DI and SI tasks directly, differential activation in several brain regions was observed. The right fusiform, bilateral superior temporal gyrus, left cuneus, right precuneus, left lingual, and right supplementary motor area were activated more strongly in the DI task than in the SI task (All cluster sizes >30 voxels, *p* < 0.05, cluster-wise FWE corrected; see Table 3; Figure 2c). In contrast, the reverse subtraction revealed that only the left cerebellum crusI lobule were activated more strongly in the SI task than in the DI task (All cluster sizes >30 voxels, *p* < 0.05, cluster-wise FWE corrected; see Table 4; Figure 2d). These differential patterns of activation suggested that while there was some overlap in the neural substrates supporting SI and DI, each task also engages unique neural processes.

**Table 3:** Brain regions activated more strongly in the dynamic imagery (DI) task than in the static imagery (SI) task.

Anatomical area	Hemi	Co-ordinates			K	*t*-value
		X	Y	Z		
Fusiform	R	40	−36	−16	2,860	4.69
Superior Temporal Gyrus	L	−50	−36	18	6,049	8.34
Cuneus	L	−16	−62	22	1,860	4.90
Superior Temporal Gyrus	R	68	−26	6	4,142	8.94
Precuneus	R	8	−46	52	7,763	7.24

Hemi, Hemisphere activation present in-left (L) or right (R). K, Cluster size. *t*-value, peak *t*-value.

**Table 4:** Brain regions activated more strongly in the static imagery (SI) task than in the dynamic imagery (DI) task.

Anatomical area	Hemi	Co-ordinates			K	*t*-value
		X	Y	Z		
Cerebellum_Crus1	L	−8	−72	−28	2,177	5.18
Undefined		−18	−10	34	9,269	8.93

Hemi, Hemisphere activation present in-left (L) or right (R). K, Cluster size. *t*-value, peak *t*-value.

### 3.4 Relationship between brain activation of bilateral precuneus and reported vividness of visual images

**Figure 3:**
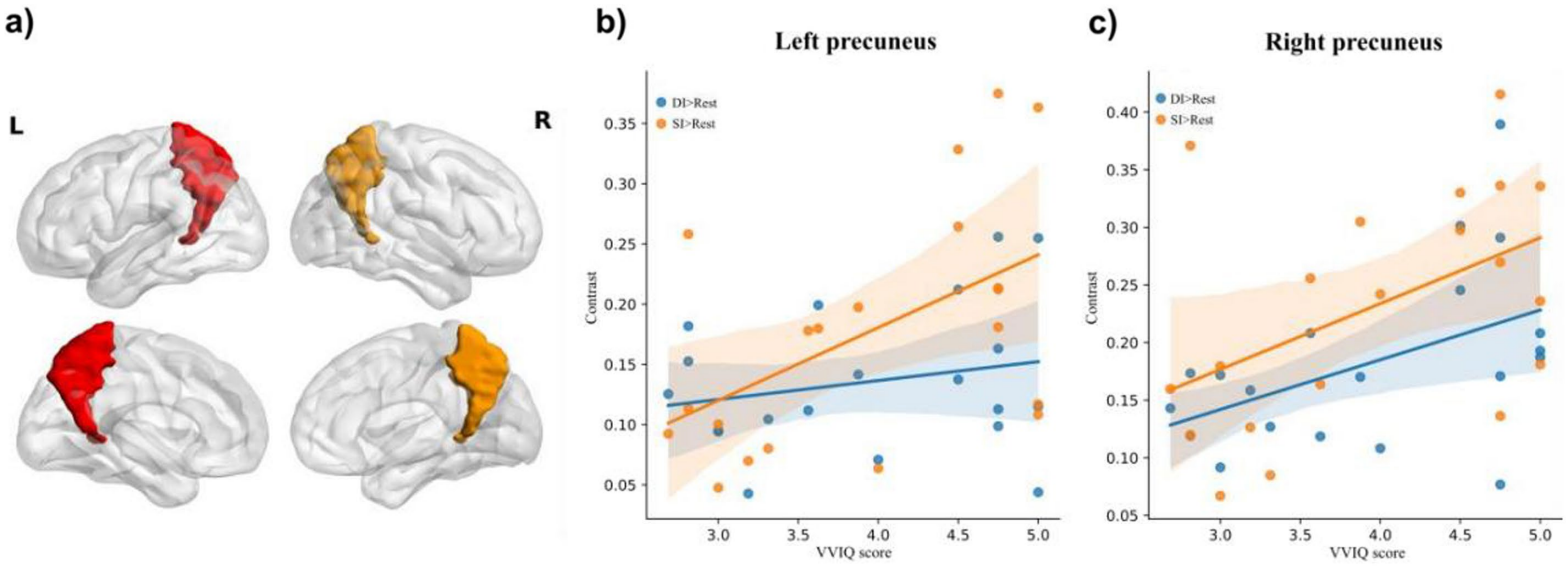
The illustration of **(a)** bilateral precuneus, and correlation of brain activation in the **(b)** and **(c)** right precuneus under dynamic imagery (DI) task (DI > Rest) and static imagery (SI) task (SI) task (SI > Rest) with the Vividness of Visual Imagery Questionnaire (VVIQ) score.

The relationship between the vividness of mental imagery, as measured by the VVIQ, and the activation of the precuneus was a key focus of our analysis. The correlations of bilateral precuneus activation with VVIQ-C scores were analyzed. For the left precuneus (Figure 3a), there was no significant correlation between activation of the DI > SI task and VVIQ score (Pearson *r* = −0.33, *p* = 0.16); no significant correlation between activation of SI > DI task and VVIQ score (Pearson *r* = 0.37, *p* = 0.11); no significant correlation between the activation of DI > Rest task and VVIQ score (Pearson *r* = 0.22, *p* = 0.35). The significant positive correlation was evident between activation of SI > Rest task and VVIQ score (Pearson *r* = 0.51, *p* = 0.02) (Figure 3b).

For the right precuneus (Figure 3a), there was no significant correlation between activation of DI > SI task and VVIQ score (Pearson *r* = 0.03, *p* = 0.91); no significant correlation between activation of SI > DI task and VVIQ score (Pearson *r* = 0.39, *p* = 0.09). Interestingly, there were significant positive associations between the VVIQ score and activation of DI > Rest task (Pearson *r* = 0.48, *p* = 0.03) and activation of SI > Rest task (Pearson *r* = 0.48, *p* = 0.03) respectively (Figure 3c). This suggests that the right precuneus may be particularly sensitive to the vividness of mental imagery, regardless of whether the imagery is static or dynamic.

## 4 Discussion

In the current study, the neural underpinnings of SI and DI in healthy participants were explored. Utilizing the Vividness of Visual Imagery Questionnaire (VVIQ-C) (Zhang et al., 2024), participants’ VVI abilities were quantified before engaging them in imagery tasks involving static and dynamic scenes. High-resolution fMRI data were acquired using a 3T scanner, and a General Linear Model (GLM) was applied to analyze BOLD signals, focusing on the precuneus’s role in imagery vividness. This methodological approach allowed us to draw correlations between subjective vividness ratings on VMI abilities and neural activations, particularly within the precuneus.

The study revealed that both SI and DI tasks were associated with activation in the precuneus, a finding that supports the precuneus’s role in VMI. The right precuneus, in particular, showed significant correlations with VVI scores during both static and DI tasks and the left precuneus showed significant correlations with VVI scores during SI task, suggesting its importance in the vividness of mental imagery. Furthermore, the DI task was found to activate more brain regions, including the precuneus, compared to the SI task.

### 4.1 Both SI and DI were associated with precuneus activation

As we predicted, our experiments revealed the left precuneus was activated in both DI > Rest task (Figure 2a) and SI > Rest task (Figure 2b), indicating a potential involvement of the precuneus in VMI. The precuneus is a region consistently implicated in a wide array of cognitive tasks, including visual–spatial imagery (Cavanna and Trimble, 2006; Mazzoni et al., 2019), memory retrieval (Hebscher et al., 2020; Mazzoni et al., 2019) and self-processing operations, namely first-person perspective taking and an experience of agency (Murray et al., 2015; Northoff et al., 2006). The current results were consistent with the observations in numerous neuroimaging studies, highlighting its role in VMI (Dijkstra et al., 2017; Fulford et al., 2018; Winlove et al., 2018). In a meta-analysis study on VMI, regions consistently activated by VMI were identified across 40 neuroimaging studies using the Activation Likelihood Estimation (ALE) algorithm which involving the activation of the precuneus (Winlove et al., 2018). Our findings, which demonstrate activation of the precuneus in both SI and DI tasks, aligned with the existing literature. For instance, the precuneus was found to be activated during SI tasks such as imagining familiar concrete objects (de Borst et al., 2012; Gardini et al., 2009; Zvyagintsev et al., 2013), has also been found to be activated in DI tasks such as mental rotation and imagining a movement (Confalonieri et al., 2012; Hanakawa et al., 2003; Podzebenko et al., 2002).

The precuneus’s activation patterns in our study are particularly noteworthy, as they reflect the brain engagement in constructing mental representations of both SI and DI. This dual involvement suggests that the precuneus may play a critical role in the core processes underlying VMI. The activation of the precuneus could be indicative of its function in integrating sensory information, spatial orientation, and self-related perspectives, which are all essential components of VMI (Cavanna and Trimble, 2006; Dadario and Sughrue, 2023; Mazzoni et al., 2019; Utevsky et al., 2014).

However, it is worth noting that the significant overlap in brain activity between SI and DI may be partially due to the nature of the experimental materials used in the SI tasks. For example, participants may have unintentionally included dynamic elements (e.g., imagining wind blowing through the forest) when visualizing static scenes. Additionally, the within-subjects design might have made the participants’ imagery in SI conditions be influenced by their exposure to DI conditions. Future studies could address this limitation by using more controlled stimuli or employing a between-subjects design to minimize such confounds.

### 4.2 Precuneus was associated with vividness of SI and DI

As predicted in our introduction, the VVIQ was significantly correlated with activation of the left precuneus in the SI task (Figure 3b), and the VVIQ was significantly correlated with activation of the right precuneus in both SI and DI tasks (Figure 3c), further supported the notion that the precuneus was not only involved in VMI but also in the vividness of the imagery experienced. The relationship between precuneus activation and the vividness of VMI, as measured by the Vividness of Visual Imagery Questionnaire (VVIQ) (Marks, 1973; Zhang et al., 2024), was a novel contribution to our study. While previous research has documented the precuneus’s activation during VMI tasks (Dijkstra et al., 2017; Fulford et al., 2018; Winlove et al., 2018), the explicit link to imagery vividness has not been thoroughly explored. Our findings revealed a significant correlation between the right precuneus activation and VVI scores in both SI and DI conditions, suggesting that this region may be a key neural substrate for the vividness of VMI.

The precuneus’s role in VMI vividness may extend beyond the realm of imagery alone. Creativity, for example, often involves the generation of novel and detailed mental images, a process that, as the previous findings suggest, may be facilitated by precuneus activation (De Pisapia et al., 2016; Gonen-Yaacovi et al., 2013). The precuneus’s engagement in tasks requiring creativity, such as artistic expression or problem-solving, further underscores its importance in cognitive functions that demand the manipulation and synthesis of complex information (Cavanna and Trimble, 2006; Dadario and Sughrue, 2023; Mazzoni et al., 2019; Utevsky et al., 2014). Moreover, the precuneus’s activation has been observed in studies on episodic memory retrieval, which shares similarities with VMI in terms of constructing mental scenes (Cavanna and Trimble, 2006; Dadario and Sughrue, 2023).

### 4.3 Precuneus is more active in DI

The present fMRI study provided intriguing insights into the neural distinctions between SI and DI, particularly highlighting the differential activation of the precuneus. Our findings indicated that DI was associated with greater activation in the right precuneus compared to SI, suggesting a more extensive neural engagement during the mental simulation of moving scenes or sequences. An existing study revealed that visual processing related regions and emotion-related regions were more active when viewing dynamic landscapes than static ones (Zhao et al., 2020).

This enhanced activation during DI aligned with previous research that has consistently reported increased neural activity during tasks requiring the manipulation of mental images (Parsons et al., 1995; Kosslyn et al., 2001). The precuneus, a region known for its role in episodic memory retrieval and visuospatial processing (Cavanna and Trimble, 2006), appears to be a common neural substrate for both SI and DI. However, our results suggest a heightened role for the right precuneus in DI, which may be attributed to its involvement in VVI. The correlation between the right precuneus activation and the vividness of VMI, as measured by the VVIQ-C, underscored the importance of this region in the clarity and detail of mental imagery. The lack of significant activation differences between SI and DI in any brain region, except for the right precuneus, indicated that while both types of imagery share common neural mechanisms, dynamic VMI may place greater demands on these mechanisms, particularly those related to the vividness of imagery. In other words, according to our finding that the right precuneus was associated with vividness in both SI and DI, we hypothesize that one of the main differences between DI and SI is the vividness of representations, i.e., DI is more vivid than SI.

In addition to the vividness factor, the enhanced activation of the right precuneus during DI may be attributed to its involvement in detailed cognitive processes like motor imagery and spatial navigation (Cavanna and Trimble, 2006; Malouin et al., 2003; Ogiso et al., 2000). During DI tasks, participants are often tasked with simulating movements or navigating through mental landscapes, which could significantly engage the right precuneus. This engagement is likely due to the precuneus’s essential function in managing spatial information and orchestrating motor actions, as highlighted by several studies (Malouin et al., 2003; Ogiso et al., 2000). Notably, the precuneus exhibits marked activation during tasks that involve imagining motion or navigating in a mental space, emphasizing its role in the dynamic components of imagery. Furthermore, the complexity of the psychological processes involved in DI may also contribute to the increased activation of the right precuneus (Hebscher et al., 2019; Jia et al., 2015; Schulz et al., 2018). Unlike SI, which primarily involves constructing a mental image of an object or scene, DI requires participants to visualize changes. DI requires more cognitive resources to simulate movement and transformation than SI, which can lead to increased activation of the right precuneus (Hebscher et al., 2019; Jia et al., 2015; Schulz et al., 2018). The need to integrate and manipulate these dynamic elements may explain why this region shows greater activation during DI tasks.

In conclusion, our fMRI study revealed the neural intricacies underlying SI and DI, with a particular emphasis on the precuneus’s role. The precuneus played a key role in the vividness of mental representations. Our findings indicated that while SI and DI share common neural substrates, the latter engages a broader network. These findings not only advance our understanding of VMI but also underscore the precuneus’s significance in shaping the vividness of mental imagery. While our study provides insights into the precuneus’s role in visual mental imagery (VMI) vividness, a key limitation is the lack of dissociation between clarity and richness of details. The Vividness of Visual Imagery Questionnaire (VVIQ) yields a composite score, making it unclear whether precuneus activation is more strongly associated with clarity or richness (Marks, 1973; Sreekumar et al., 2018). Future studies could address this by incorporating separate ratings for clarity and richness during imagery tasks, enabling correlation analyses to identify distinct neural substrates. Additionally, seed-based functional connectivity analysis could elucidate the precuneus’s contributions to clarity versus richness (Fulford et al., 2018; Saiote et al., 2016). Finally, developing objective measures for clarity and richness remains a challenge. Emerging techniques such as multivariate pattern analysis (MVPA), machine learning, and neurofeedback could provide more quantifiable and dynamic assessments of these aspects, advancing our understanding of VMI vividness (Dijkstra et al., 2019; Winlove et al., 2018).

## Data Availability

The raw data supporting the conclusions of this article will be made available by the authors, without undue reservation.

## Footnotes

^1^ www.fil.ion.ucl.ac.uk/spm

^2^ https://gitee.com/bmeqingfenglee/vviq
